# Transvaginal Uterine Fibroid Radiofrequency Ablation (TV-RFA): Retrospective Analysis and Preliminary Report

**DOI:** 10.3390/life15121841

**Published:** 2025-11-30

**Authors:** Karolina Chmaj-Wierzchowska, Agnieszka Lach, Kinga Bednarek, Adrian Nowak, Adrian Mruczyński, Alan Bruszewski, Piotr Piekarski, Adam Malinger, Maciej Wilczak

**Affiliations:** Department of Maternal and Child Health and Minimally Invasive Surgery, Poznan University of Medical Sciences, 60-701 Poznan, Poland; lach.agnieszka95@gmail.com (A.L.); kinga.bednarek88@gmail.com (K.B.); adriannowakk@wp.pl (A.N.); adrianmrucz@gmail.com (A.M.); abruszewski@ump.edu.pl (A.B.); piotr.piekarski@ump.edu.pl (P.P.); adam.malinger@ump.edu.pl (A.M.); mwil@ump.edu.pl (M.W.)

**Keywords:** transvaginal radiofrequency ablation (TV-RFA), uterine fibroids, menstrual bleeding

## Abstract

(1) Background: Transvaginal RFA is a minimally invasive treatment for myomas in women opting for uterus preservation. The present study aimed to evaluate the efficacy and safety of transvaginal RFA to treat myomas, reduce symptoms, decrease myoma volume, and identify prognostic factors for predicting treatment response. (2) Methods: The study group included 45 women treated for uterine fibroids at the Gynecological and Obstetrics Clinical Hospital in Poznań. From 1 July 2024 to 31 March 2025, a total of 45 transvaginal radiofrequency ablation (TV-RFA) procedures were performed. (3) Results: Ultrasound findings revealed that fibroid dimensions and volume significantly decreased at 1-month follow-up compared to those at pre-procedure (88.7 ± 116.3 vs. 64.6 ± 82.6 cm^3^; *p* = 0.003). Ultrasound findings demonstrated that fibroid depth (4.8 ± 2.1 vs. 4.1 ± 2.2; *p* = 0.01) and fibroid volume (88.7 ± 116.3 vs. 82.4 ± 93.9 cm^3^; *p* = 0.02) were significantly decreased at 3-month follow-up compared to their pre-procedure values. Menstrual bleeding duration showed significant differences between the pre-procedure state and 1-month follow-up (N = 23; T = 2; Z = 4.14; *p* < 0.001) and 3-month follow-up (N = 8; T = 1; Z = 2.38; *p* = 0.017), with a significant reduction after the RFA procedure. Significant differences were observed in bleeding severity at pre-procedure and at 1-month follow-up (N = 22; T = 11.5; Z = 3.73; *p* < 0.001); however, no significant differences in bleeding severity were noted at the 3-month follow-up (N = 7; T = 4; Z = 1.69; *p* = 0.09). These results should be interpreted cautiously due to the small number of patients with complete 3-month follow-up. (4) Conclusions: Transvaginal radiofrequency ablation is an effective, precise, and safe minimally invasive approach for treating uterine fibroids. These preliminary findings are promising but require confirmation in larger cohorts with longer follow-up.

## 1. Introduction

Uterine fibroids are the predominant pelvic benign neoplasms occurring in reproductive-aged women and the leading benign indication for undergoing hysterectomy [1]. The incidence rate of fibroids is 2.0–9.2 per 1000 woman-years, and the incidence increases with age until menopause [2,3]. While fibroids are often asymptomatic, symptoms such as abnormal uterine bleeding, pelvic pressure, pain, subfertility, and dyspareunia can help diagnose fibroids [1]. Heavy menstrual bleeding may be caused by submucous fibroids (FIGO types 0–2) and intramural fibroids (FIGO types 3–5) [4,5]. Subserosal fibroids (FIGO types 6–7) are frequently asymptomatic until they become large enough to induce bulk symptoms. Transmural fibroids have submucosal and subserosal components.

Traditional treatments for symptomatic fibroids include major surgeries such as hysterectomy and myomectomy, which are associated with significant complications and high morbidity [6]. Moreover, hysterectomy does not preserve the uterus and fertility; consequently, this surgical approach is not feasible for women planning for future pregnancies or for those who wish to retain their uterus for other reasons. Furthermore, although myomectomy can be performed through laparotomy, laparoscopy, hysteroscopy, or transvaginal approach, it does not serve as a definitive therapy in many cases.

In the last few years, researchers have proposed some alternative minimally invasive approaches to treat symptomatic uterine fibroids to avoid major surgery and preserve potential fertility [7,8]. Uterine artery embolization (UAE) is reported to be a safe and effective treatment for hypervascular fibroids [9], although fertility-related issues associated with this approach remain debatable [10]. Currently, the UAE is not recommended for women who want to preserve fertility; moreover, there is a possibility of fibroid recurrence in women receiving UAE, with approximately 20% of patients subsequently requiring hysterectomy [11]. In recent years, new minimally invasive techniques have been adopted for treating uterine fibroids and have improved the quality of life of treated women. These procedures use various forms of energy to heat and ablate uterine fibroids, including radiofrequency energy, focused ultrasound, and microwaves [12,13,14]. Radiofrequency ablation (RFA) is a widely used modality to achieve the local control of fibroids; this technique produces thermal fixation and coagulation necrosis within the fibroids, leading to a reduction in fibroid volume and a decrease in or elimination of symptoms related to uterine fibroids [15].

Compared with other conservative approaches—such as UAE, MR-guided focused ultrasound (MRgFUS), and laparoscopic RFA—transvaginal RFA provides direct access to intramural and submucosal components, enabling precise, real-time ultrasound-guided ablation without abdominal incisions or vascular occlusion. This route minimizes operative trauma and reduces recovery time while avoiding radiation exposure. Despite these advantages, current evidence remains limited to selected cohorts, and long-term real-world data are still scarce.

Transvaginal RFA is a minimally invasive treatment for myomas in women opting for uterus preservation. The present study aimed to evaluate the efficacy and safety of transvaginal RFA to treat myomas, reduce symptoms, decrease myoma volume, and identify prognostic factors for predicting treatment response. Transvaginal radiofrequency ablation appears to be an effective and safe minimally invasive method for treating uterine fibroids. Given the short follow-up period and the limited number of patients with complete 3-month data, the present results should be considered preliminary and interpreted with caution.

## 2. Materials and Methods

The study group included 45 women treated for uterine fibroids at the Gynecological and Obstetrics Clinical Hospital in Poznań. From 1 July 2024 to 31 March 2025, a total of 45 transvaginal radiofrequency ablation (TV-RFA) procedures were performed.

This study was a retrospective analysis of anonymized clinical data obtained during routine clinical care. Because the analysis includes early cases from an ongoing cohort, follow-up duration varied, and only a subset of patients had completed the 3-month evaluation at the time of data lock.

### 2.1. Inclusion and Exclusion Criteria

Patients were considered suitable candidates for RFA of uterine fibroids if they met the following inclusion criteria:

1. Symptomatic intramural fibroids (up to three fibroids) according to the International Federation of Gynecologists and Obstetricians (FIGO) classification types 3–4, with a maximum diameter of 8 cm, in cases where the following applies:
  - the patient had contraindications to surgical treatment using other techniques,
  - the patient did not consent to surgical treatment using other techniques, or
  - previous pharmacological therapies were ineffective in alleviating symptoms.
2. Symptomatic submucosal fibroids (manifesting as heavy menstrual bleeding) according to the FIGO classification types 0–2, only when previous hysteroscopic myomectomy had been ineffective.
3. Symptomatic subserosal fibroids (causing pain and urinary or bowel disorders) according to the FIGO classification types 5–6, when previous surgical treatment was ineffective or contraindicated.

To ensure procedural safety and efficacy, the following exclusion criteria were considered to identify patients who may not be suitable candidates to undergo RFA of uterine myomas (fibroids):

1. Patients with submucosal fibroids according to FIGO classification types 0–2 (except when hysteroscopic myomectomy had been ineffective) or subserosal fibroids according to FIGO classification types 5–6 (except when surgical treatment was ineffective or contraindicated).
2. Patients with pedunculated fibroids or fibroids in other locations, according to FIGO classification types 7–8.
3. Suspicion of sarcoma or atypical fibroid on magnetic resonance imaging (MRI).
4. Patients with malignancy of the reproductive organs, cervical dysplasia, vaginal/cervical or pelvic infection, or severe systemic diseases.
5. Patients planning future pregnancies.

In patients showing heavy or abnormal uterine bleeding, neoplastic transformations were excluded based on endometrial and/or endocervical canal biopsy performed under local anesthesia during hysteroscopy. All patients underwent contrast-enhanced pelvic MRI within 6 months prior to the procedure to exclude atypical lesions.

Preoperative investigations included clinical evaluation of the symptoms and ultrasound examination to determine the number, position, and size of fibroids and the distance between the fibroid pseudocapsule and uterine serosa. Blood count, coagulation assay, and preoperative evaluation were also conducted.

All ultrasound examinations were performed by the same experienced operators using the same ultrasound unit and identical imaging settings to minimize inter-operator and inter-device variability.

All patients were initially informed about the efficacy, risks, and benefits of the RFA technique, and written informed consent to undergo this procedure was subsequently obtained from them. Patient consent for inclusion in the retrospective study was waived by the Bioethics Committee because only anonymized clinical data were analyzed

### 2.2. Ultrasound Examination

Before uterine fibroid ablation, patients underwent a presurgical analysis, including blood work and electrocardiography. Prior to treatment, an ultrasound scan was performed to obtain an accurate assessment of the number, dimension, and location of uterine fibroids and classify them into one of four groups (submucosal, intramural, subserosal, or hybrid) based on the FIGO system. The mean diameter and volume of each fibroid were calculated by ultrasound using the following formulas: Mean diameter = (Length + Width + Height)/3, and for volume reduction calculations used in the Results, fibroid volume was consistently determined using the standard ellipsoid formula: Volume = (4/3) × π × (Length/2 × Width/2 × Depth/2)

### 2.3. Description of the RFA System

A VIVA RF System (STARmed) radiofrequency generator was utilized to create an ablation zone. This system is equipped with a 17 G RF fixed coagulation needle electrode (length: 35 cm, reference number: 17−35 s 30 F) with a 1-cm active tip. The circuit is cooled with a continuous infusion pump. The generator operates at 480 kHz and heats the electrode at the needle tip to temperatures between 60 °C and 90 °C. This intense temperature induces protein degradation within the fibroid through coagulation necrosis. Although the generator has a maximum power of 200 W, we used 35 W, as the maximum power for all procedures in our study.

### 2.4. Description of the Transvaginal RFA Procedure

The patients were intravenously administered a first-generation cephalosporin (2 g) as a prophylactic treatment. Transvaginal RFA was then performed under general anesthesia in an outpatient setting, with patients placed in a dorsal position on an empty bladder. We adopted standard sterile techniques and preparative procedures during the RFA process. Under ultrasound guidance, the needle electrode was inserted through the anterior or posterior fornix of the vagina until the needle tip was located within the fibroid, 0.5 cm deep to the pseudocapsule. In each ablation shot, the power released and tissue impedance changes were monitored through the radiofrequency generator screen, and a permanent change in the echogenicity of the treated tissue (i.e., becoming hyperechoic) was confirmed through the ultrasound image. After treating each target area, the needle was moved to an adjacent, untreated part of the fibroid, and the ablation process was continued. The process was considered complete when approximately 80% of the fibroid volume showed a change in echogenicity. Appropriate safety measures were implemented to avoid thermal damage to the surrounding healthy tissues. Following treatment completion, vaginal and cervical bleeding were assessed, and bladder catheterization was performed. The patient was discharged home approximately 5 h after the procedure, unless additional indications required continued hospitalization. Patients were recommended to take non-steroidal anti-inflammatory drugs as needed for pain control. The following postoperative medications were prescribed: oral second-generation cephalosporin, 1 tablet twice daily for 5 days; vaginal suppositories with policresulen, 1 suppository every alternate day; and rectal diclofenac, 1 suppository twice daily for 5 days. Postoperative analgesia followed a standardized NSAID-based protocol. Patients were discharged once hemodynamically stable, ambulatory, without significant vaginal bleeding, and reporting pain ≤ 3/10.

### 2.5. Statistical Analysis

Statistical Analysis: Data distribution was evaluated using the Shapiro–Wilk test. Due to non-normal distribution and small sample sizes, non-parametric tests (Wilcoxon signed-rank test, Mann–Whitney U test, chi-square or Fisher’s exact tests) were used. Paired analyses included only patients with complete pre- and post-procedure measurements. Missing data were handled using pairwise deletion. Given the exploratory nature of this preliminary study, no correction for multiple comparisons was applied; therefore, results should be interpreted cautiously.

## 3. Results

Forty-five patients with uterine fibroids were included in the study. Because follow-up completeness varied across the cohort, subsequent analyses (Section 3.2, Section 3.3, Section 3.4, Section 3.5, Section 3.6, Section 3.7 and Section 3.8) are based on different numbers of patients depending on data availability.

### 3.1. Patient Characteristics

The mean age of the patients was 43.7 ± 5.4 years (range: 32–53 years). Twenty-one patients (46.67%) were aged ≤44 years, and 24 patients (53.33%) were aged >45 years. The mean body weight was 71.6 ± 14.6 kg (range: 48–100 kg), and the mean height was 166.7 ± 5.9 cm (range: 153–178 cm). The mean body mass index (BMI) was 25.7 ± 4.5 kg/m^2^ (range: 18–35.7 kg/m^2^). Table 1 shows the characteristics of the study patients.

### 3.2. Fibroids

Thermal ablation was performed on intramural fibroids (N = 41), with some fibroids located mainly in the anterior wall (N = 23). Table 2 shows the characteristics of the detected uterine fibroids. These baseline distribution data reflect the full cohort (N = 45); however, subsequent analyses involving fibroid measurements are based on smaller numbers of patients due to incomplete follow-up. The predominance of Funaki II lesions is relevant for the interpretation of subgroup effects.

### 3.3. Data Analysis

#### 3.3.1. Obstetric Data

As shown in Table 3, the number of deliveries exhibited no correlation with the Funaki type, patient’s age, or body weight assessment. Because subgroup numbers were small in several categories, these analyses should be interpreted with caution, as the study may have been underpowered to detect weaker associations.

Patients with Funaki type II fibroids, those with age > 45 years, and those showing normal body weight had a significantly higher incidence of miscarriages, particularly in patients with three miscarriages (Table 4).

#### 3.3.2. Hormonal Therapy

Within the study group, patients with abnormal body weight used contraceptive medications significantly more frequently than those with normal body weight (Table 5).

### 3.4. Fibroid Localization Detected by Ultrasound

Ultrasound findings revealed that anterior localization of uterine fibroids was significantly higher in patients with age > 45 years, whereas posterior localization of uterine fibroids was significantly more common in patients with age < 45 years (Table 6). These localization patterns are based on small subgroup numbers, and although statistically significant, they should be interpreted carefully.

The classification of uterine fibroids (intramural, submucosal, or subserosal) based on ultrasound findings showed no correlation with Funaki type, age, body weight assessment, or history of delivery (Table 7).

### 3.5. Procedure Time

The procedure time among patients ranged from 10 to 30 min. The average procedure time for the entire study group was 16 min (17.2 ± 4.5 min). As shown in Table 8, no correlation was observed between procedure time and Funaki type, patient’s age, body weight assessment, and history of delivery.

### 3.6. Patient Follow-Up

Table 9 shows the results of patient follow-up at 1 month after the procedure. Ultrasound findings revealed that fibroid dimensions and volume significantly decreased at 1-month follow-up compared to those at pre-procedure (88.7 ± 116.3 vs. 64.6 ± 82.6 cm^3^; *p* = 0.003).

Table 10 presents the results of patient follow-up at 3 months after the procedure. Ultrasound findings demonstrated that fibroid depth (4.8 ± 2.1 vs. 4.1 ± 2.2; *p* = 0.01) and fibroid volume (88.7 ± 116.3 vs. 82.4 ± 93.9 cm^3^; *p* = 0.02) were significantly decreased at 3-month follow-up. The effect observed at 3 months was less pronounced than at 1 month, likely reflecting the small number of patients with complete 3-month follow-up (N = 10) and early-stage variability in post-ablation remodeling.

### 3.7. Treatment Effect

The effects of RFA treatment on patients at 1 and 3 months after the procedure are shown in Table 11.

Patients with Funaki type II fibroids showed a significantly greater treatment effect at 1-month follow-up, with a significant decrease in fibroid length, width, depth, and volume (Table 12). This pattern is consistent with previous reports suggesting that Funaki II lesions respond more uniformly to thermal ablation.

Patients with age < 45 years and age > 45 years showed no significant differences in treatment effects (Table 13).

Furthermore, patients with normal body weight exhibited a significantly greater treatment effect at the 3-month follow-up, with ultrasound measurements showing a significant decline in fibroid depth alone (Table 14). Because only ten patients had completed the 3-month follow-up, these results should be interpreted very cautiously.

The treatment effect did not differ significantly between patients with differences in the history of delivery (Table 15). This analysis was based on very small subgroups, limiting the ability to detect potential associations. Because of the small sample size in each subgroup, particularly for 3-month comparisons, these findings should be interpreted cautiously.

### 3.8. Effect of RFA Treatment on Menstruation

**Figure 1:**
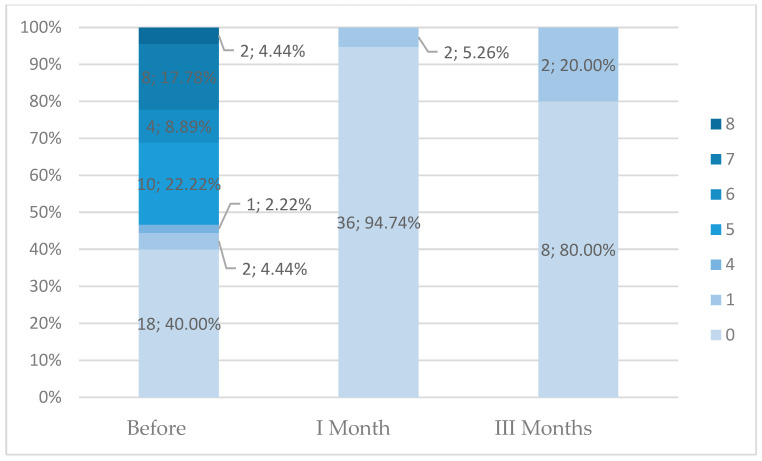
Bleeding duration at the pre-procedure state and at 1-month and 3-month follow-up.

Figure 1 depicts bleeding duration among the patients. Bleeding duration showed significant differences between the pre-procedure state and 1-month follow-up (N = 23; T = 2; Z = 4.14; *p* < 0.001) and 3-month follow-up (N = 8; T = 1; Z = 2.38; *p* = 0.017), with a significant reduction after the RFA procedure. These results are based on small paired samples, particularly at 3 months (N = 8), and therefore must be interpreted with caution.

Bleeding duration also exhibited no correlation with Funaki type, patient’s age, or history of delivery at 1-month follow-up after RFA treatment. However, patients with normal body weight reported bleeding significantly more frequently before the procedure (Table 16). These findings are based on small subgroup numbers, and therefore, the absence or presence of associations should be interpreted cautiously.

**Figure 2:**
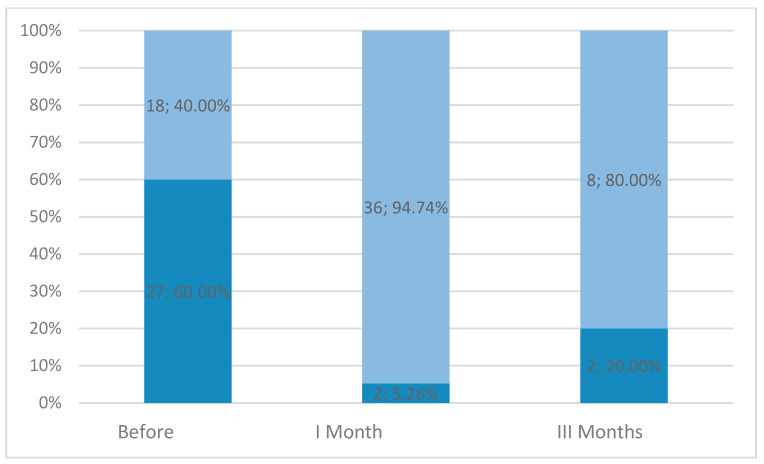
Bleeding severity at the pre-procedure state and at 1-month and 3-month follow-up (▪ NO, ▪ YES).

Figure 2 shows the number of patients experiencing heavy menstruation. Significant differences were observed in bleeding severity at pre-procedure and at 1-month follow-up (N = 22; T = 11.5; Z = 3.73; *p* < 0.001); however, no significant differences in bleeding severity were noted at the 3-month follow-up (N = 7; T = 4; Z = 1.69; *p* = 0.09).

No correlations were observed between bleeding occurrence and the patient’s age, body weight assessment, and Funaki type.

### 3.9. Complications

None of the patients experienced any complications according to the Clavien–Dindo Classification of Surgical Complications. However, given the limited sample size and short follow-up, rare complications cannot be fully excluded.

## 4. Discussion

Transvaginal thermoablation of uterine fibroids involves the destruction of fibroid tissues by using high temperatures generated by radiofrequency waves (RFA) without requiring skin or abdominal wall incisions. This technique facilitates the treatment of submucosal fibroids located within the uterine cavity, intramural fibroids with a submucosal component, and certain intramural fibroids without endometrial contact. Based on precise guidance with an intrauterine ultrasound probe, this procedure is safe, effective, and minimally invasive [16,17,18,19].

The efficacy of transvaginal thermoablation has been confirmed in the Fibroid Ablation Study—Europe (FAST-EU) [20] and the Sonography-Guided Transcervical Ablation of Uterine Fibroids (SONATA Trial) [21] as well as in several observational-retrospective studies reported by Santalla-Hernández et al. [16,17,18,19]. FAST-EU, one of the first large-scale clinical studies to evaluate transvaginal thermoablation, confirmed that this procedure is both effective and adequately tolerated by patients. At 12 months post-procedure, more than 90% of patients showed significant improvements in symptoms, particularly menorrhagia and menstrual pain. The average reduction in fibroid volume was approximately 45–60% within 3–6 months of treatment. The majority of patients were satisfied with the treatment outcomes and did not require further surgical intervention within 1 year after treatment. The average period of return to daily activities was 3 days [20].

The SONATA Trial, a key multicenter study conducted in the United States, evaluated the safety and efficacy of transvaginal thermoablation. Within the first 12 months of treatment, 95% of patients showed a significant reduction in fibroid-related symptoms. Three years after treatment, more than 85% of patients did not require additional surgical or pharmacological treatment. Data from UFS-QoL questionnaires revealed substantial improvements in patients’ quality of life (QoL), with no occurrence of severe complications; based on these findings, the procedure was considered safe with a minimal risk [21]. The observational data of studies conducted by Santalla-Hernández et al. [16,17,18,19] also confirmed the high efficacy of this treatment method, with an average reduction of 40–60% in fibroid size within 6 months of treatment. The frequently reported benefits included improved QoL, disappearance of lower abdominal pain and pressure symptoms, shorter menstrual duration, reduced bleeding severity by more than 50%, and high patient satisfaction. Furthermore, over 92% of patients did not require further treatment for 24 months after thermoablation [16,17,18,19].

In the present study, we noted a significant decrease in fibroid dimensions and volume at both 1- and 3-month follow-ups compared to those at the pre-procedure state. Moreover, bleeding duration and intensity were also significantly decreased after transvaginal thermoablation. Patients with symptomatic fibroids larger than 8 cm were also eligible to undergo this procedure. Additionally, the reduction in fibroid size after transvaginal thermoablation allowed subsequent safe surgical treatment in three patients. However, these findings should be interpreted cautiously because only a subset of patients had complete 1-month data, and only 10 patients completed the 3-month follow-up, limiting the strength of the conclusions. The less pronounced effect at 3 months may reflect both the small number of complete follow-up cases and the early phase of biological remodeling, during which edema resolution and tissue contraction may occur at variable rates.

Clinical data indicate a low complication rate for transvaginal thermoablation. Most complications are mild, such as bleeding/spotting for a few days post-procedure, lower abdominal pain, and increased vaginal discharge. On rare occasions, patients may develop infection or sustain perforation or thermal injury to surrounding tissues (bladder or intestines) [16,17,18,19]. In the present study, none of the patients developed complications classified according to the Clavien–Dindo system.

Patients planning pregnancy in the future were initially excluded from receiving RFA. However, recent data suggest that this procedure may also be safe for women planning for pregnancy [22]. Martínez et al. [22] reported that among patients attempting to conceive within 24 months of treatment, 73.68% (14/19) achieved successful childbirth. There were no cases of uterine rupture, preterm birth, or intrauterine fetal death [22].

In our cohort, patients with Funaki II fibroids demonstrated greater volume reduction after 1 month. This may be related to the intermediate stromal density and vascularity characteristic of Funaki II lesions, which could allow more uniform heat propagation and more effective coagulation necrosis.

Thus, transvaginal thermoablation of uterine fibroids is a modern, effective, and safe alternative to traditional surgical therapy. It is particularly suitable for women desiring to preserve their uterus, avoid surgery, or quickly return to daily activities. The decision to undergo this treatment should be personalized based on the patient’s current and future conditions, proper consultation with a gynecologist, and thorough imaging diagnostics. Future studies with larger cohorts, standardized imaging follow-up, and longer observation periods are needed to validate these preliminary findings.

## 5. Conclusions

Transvaginal thermoablation is an effective, precise, and safe minimally invasive approach for treating uterine fibroids. Based on the available short-term data, the procedure appears to be most effective for small and medium-sized fibroids; however, this observation should be interpreted cautiously, as a size-stratified analysis was not performed. These findings are preliminary and require confirmation in larger prospective cohorts with longer follow-up to determine the durability of treatment effects and the influence of fibroid characteristics on outcomes.

## Figures and Tables

**Table 1:** Characteristics of the Study Patients.

		N = 45 (%)
Age	<44 years	21 (46.67)
	>45 years	24 (53.33)
BMI (kg/m^2^)	Underweight (BMI < 18.49 kg/m^2^)	1 (2.22)
	Normal BMI (BMI 18.5–24.99 kg/m^2^)	20 (44.44)
	Overweight (BMI 25.0–29.99 kg/m^2^)	17 (37.78)
	Obesity (>BMI 30.0 kg/m^2^)	7 (15.56)
Number of Deliveries	No deliveries	18 (40)
	1 delivery	9 (20)
	2 deliveries	11 (24.44)
	3 deliveries	6 (13.33)
	4 deliveries	1 (2.22)
Number of Miscarriages	No miscarriages	37 (82.22)
	1 miscarriages	2 (4.44)
	2 miscarriages	1 (2.22)
	3 miscarriages	5 (11.11)
Hormonal Medication	No	35 (77.78)
	Yes—hormonal contraception	7 (15.56)
	Yes—Relugolix-Estradiol -Norethisterone Acetate	3 (6.67)

**Table 2:** Characteristics of the detected uterine fibroids.

		N = 45 (%)
Type of fibroids	Intramural	41 (91.11)
	Subserosal	2 (4.44)
	Submucosal	2 (4.44)
Location	Anterior wall	23 (51.11)
	Posterior wall	13 (28.89)
	Fundus	7 (15.56)
	Cavity	2 (2.22)
Funaki type	Funaki I	21 (46.67)
	Funaki II	24 (53.33)

**Table 3:** Relationship between the number of deliveries, Funaki type, age, and body weight assessment.

		Number of Deliveries					*χ* ^2^	*p*
		0	1	2	3	4		
Funaki Type	I	10 (47.62%)	5 (23.81%)	4 (19.05%)	2 (9.52%)	0 (0%)	3.03	0.55
	II	8 (33.33%)	4 (16.67%)	7 (29.17%)	4 (16.67%)	1 (4.17%)		
Age	<45 years	10 (47.62%)	5 (23.81%)	2 (9.52%)	3 (14.29%)	1 (4.76%)	6.34	0.18
	>45 years	8 (33.33%)	4 (16.67%)	9 (37.5%)	3 (12.5%)	0 (0%)		
Body weight assessment	Normal	6 (30%)	6 (30%)	5 (25%)	2 (10%)	1 (5%)	4.76	0.31
	Abnormal	12 (50%)	3 (12.5%)	5 (20.83%)	4 (16.67%)	0 (0%)		

**Table 4:** Relationship between the number of miscarriages and Funaki type, age, and body weight assessment.

		Number of Miscarriages				*χ* ^2^	*p*
		0	1	2	3		
Type	I	18 (85.71%)	2 (9.52%)	1 (4.76%)	0 (0%)	10.92	0.01
	II	19 (79.17%)	0 (0%)	0 (0%)	5 (20.83%)		
Age	<45 years	19 (90.48%)	1 (4.76%)	1 (4.76%)	0 (0%)	8.14	0.04
	>45 years	18 (75%)	1 (4.17%)	0 (0%)	5 (20.83%)		
Body weight assessment	Normal	13 (65%)	1 (5%)	1 (5%)	5 (25%)	10.77	0.01
	Abnormal	23 (95.83%)	1 (4.17%)	0 (0%)	0 (0%)		
Delivery	Yes	20 (74.07%)	1 (3.7%)	1 (3.7%)	5 (18.52%)	6.75	0.08
	No	17 (94.44%)	1 (5.56%)	0 (0%)	0 (0%)		

**Table 5:** Details of hormonal medications.

		Hormonal Medication			*χ* ^2^	*p*
		None	Contraceptives	Relugolix-Estradiol -Norethisterone Acetate		
Type	I	16 (76.19%)	2 (9.52%)	3 (14.29%)	5.54	0.06
	II	19 (79.17%)	5 (20.83%)	0 (0%)		
Age	<45 years	17 (80.95%)	4 (19.05%)	0 (0%)	4.13	0.13
	>45 years	18 (75%)	3 (12.5%)	3 (12.5%)		
Body weight assessment	Normal	18 (90%)	0 (0%)	2 (10%)	9.8	0.01
	Abnormal	16 (66.67%)	7 (29.17%)	1 (4.17%)		
Delivery	Yes	21 (77.78%)	4 (14.81%)	2 (7.41%)	0.08	0.96
	No	14 (77.78%)	3 (16.67%)	1 (5.56%)		

**Table 6:** Uterine fibroid localization based on ultrasound findings.

		Ultrasound Localization					*χ* ^2^	*p*
		Fundus	Posterior	Anterior	Cavity	Cervical		
Type	I	3 (14.29%)	5 (23.81%)	11 (52.38%)	1 (4.76%)	1 (4.76%)	3.46	0.48
	II	4 (16.67%)	8 (33.33%)	12 (50%)	0 (0%)	0 (0%)		
Age	<45 years	3 (14.29%)	10 (47.62%)	7 (33.33%)	0 (0%)	1 (4.76%)	10.31	0.04
	>45 years	4 (16.67%)	3 (12.5%)	16 (66.67%)	1 (4.17%)	0 (0%)		
Body weight assessment	Normal	3 (15%)	4 (20%)	11 (55%)	1 (5%)	1 (5%)	3.95	0.41
	Abnormal	4 (16.67%)	8 (33.33%)	12 (50%)	0 (0%)	0 (0%)		
Delivery	Yes	4 (14.81%)	7 (25.93%)	15 (55.56%)	1 (3.7%)	0 (0%)	3.35	0.5
	No	3 (16.67%)	6 (33.33%)	8 (44.44%)	0 (0%)	1 (5.56%)		

**Table 7:** Relationship between ultrasound-based classification of uterine fibroids and Funaki type, age, body weight assessment, and history of delivery.

		Ultrasound (USG) Localization			*χ* ^2^	*p*
		Intramural	Submucosal	Subserosal		
Type	I	20 (95.24%)	1 (4.76%)	0 (0%)	2.6	0.27
	II	21 (87.5%)	1 (4.17%)	2 (8.33%)		
Age	<45 years	20 (95.24%)	1 (4.76%)	0 (0%)	2.6	0.27
	>45 years	21 (87.5%)	1 (4.17%)	2 (8.33%)		
Body weight assessment	Normal	17 (85%)	1 (5%)	2 (10%)	3.31	0.19
	Abnormal	23 (95.83%)	1 (4.17%)	0 (0%)		
Delivery	Yes	24 (88.89%)	1 (3.7%)	2 (7.41%)	2.16	0.34
	No	17 (94.44%)	1 (5.56%)	0 (0%)		

**Table 8:** Relationship between procedure time and Funaki type, patient’s age, body weight assessment, and history of delivery.

Procedure Time		N Valid	M ± SD	Min–Max	Me [Q1–Q3]	*U*	*p*
Type	I	21	17.7 ± 4.3	10–25	18 [15–20]	202	0.25
	II	24	16.8 ± 4.76	12–30	15 [15–17]		
Age	<45 years	21	16.1 ± 3.8	10–25	16 [12–18]	202.5	0.26
	>45 years	24	18.2 ± 4.9	12–30	16 [15–20]		
Body weight assessment	Normal	20	16.8 ± 2.67	12–22	15 [15–20]	238.5	0.98
	Abnormal	24	17.7 ± 5.7	10–30	17 [12.5–20]		
Delivery	Yes	27	17.3 ± 4.6	12–30	15 [15–18]	237.5	0.91
	No	18	17.1 ± 4.5	10–25	17.5 [12–20]		

**Table 9:** Ultrasound findings at 1-month follow-up.

Ultrasound (USG)	Follow-Up					
	Before			1 Month		
	M ± SD	Min–Max	Me [Q1–Q3]	M ± SD	Min–Max	Me [Q1–Q3]
A. Length	4.8 ± 2	2–9.5	4.5 [3.1–6]	4.5 ± 1.9	2–9.5	4.25 [3–5.6]
B. Width	4.7 ± 2.2	2–11.5	4.17 [3.1–6.3]	4.1 ± 1.9	1–9	3.9 [2.8–5.2]
C. Depth (3rd dimension)	4.8 ± 2.1	2–10.7	4.4 [3.2–5.9]	4.1 ± 2	1–8.5	3.95 [2.7–5.2]
D. Volume	88.7 ± 116.3	4–572.3	43.46 [16.3–114.7]	64.6 ± 82.6	2–330	36.42 [11.3–81.5]

Wilcoxon test results: A. N = 34; T = 175; Z = 2.09; *p* = 0.04. B. N = 35; T = 156; Z = 2.6; *p* = 0.01. C. N = 38; T = 111.5; Z = 3.76; *p* < 0.001. D. N = 37; T = 157; Z = 2.93; *p* = 0.003.

**Table 10:** Ultrasound findings at 3-month follow-up.

Ultrasound (USG)	Follow-Up					
	Before			3 Months		
	M ± SD	Min–Max	Me [Q1–Q3]	M ± SD	Min–Max	Me [Q1–Q3]
A. Length	4.8 ± 2	2–9.5	4.5 [3.1–6]	5.3 ± 2.5	2–9.9	4.85 [3–6.9]
B. Width	4.7 ± 2.2	2–11.5	4.17 [3.1–6.3]	4.6 ± 2.1	1–7.7	4.2 [3.4–6.9]
C. Depth (3rd dimension)	4.8 ± 2.1	2–10.7	4.4 [3.2–5.9]	4.1 ± 2.2	1–7.5	3.9 [2.9–5.9]
D. Volume	88.7 ± 116.3	4–572.3	43.46 [16.3–114.7]	82.4 ± 93.9	1–250.7	40.11 [21.5–147.2]

Wilcoxon test results: A. N = 10; T = 24; Z = 0.36; *p* = 0.72. B. N = 10; T = 14; Z = 1.38; *p* = 0.17. C. N = 10; T = 1; Z = 2.7; *p* = 0.01. D. N = 10; T = 5; Z = 2.29; *p* = 0.02.

**Table 11:** Treatment effects based on ultrasound measurement.

Measurement	Follow-Up	N	M ± SD	Min–Max	Me [Q1–Q3]
USG length	Before-1 month	38	−0.2 ± 0.8	−2–1.5	−0.27 [−0.6–0]
	Before-3 months	10	−0.2 ± 1	−2–1.3	−0.06 [−0.4–0.4]
USG width	Before-1 month	38	−0.4 ± 1.1	−5–1.9	−0.37 [−0.6–0.1]
	Before-3 months	10	−0.4 ± 0.7	−2–0.6	−0.18 [−0.8–0]
USG 3rd dimension	Before-1 month	38	−0.5 ± 0.8	−3–1.5	−0.48 [−0.9–−0.2]
	Before-3 months	10	−1.2 ± 1	−3–0	−1.13 [−1.8–−0.5]
USG volume	Before-1 month	38	−17.9 ± 67	−377–124.7	−4.83 [−23.7–−1]
	Before-3 months	10	−24.7 ± 26.1	−79–8	−27.89 [−37.5–−2.2]

**Table 12:** Relationship between Funaki type and treatment effect based on ultrasound measurements.

	Type						*U*	*p*
	I			II				
	M ± SD	Min–Max	Me [Q1–Q3]	M ± SD	Min–Max	Me [Q1–Q3]		
IA	0.2 ± 0.7	−1–1.5	0 [−0.3–0.5]	−0.6 ± 0.71	−2.1–1.3	−0.48 [−0.7–−0.2]	73	0.002
IB	0.1 ± 0.8	−1–1.9	0 [−0.5–0.5]	−0.8 ± 1.17	−4.8–0.3	−0.5 [−0.9–−0.1]	97	0.02
IC	−0.1 ± 0.7	−1–1.5	−0.29 [−0.6–0.2]	−0.8 ± 0.7	−3.2–0	−0.68 [−0.9–−0.3]	99.5	0.02
ID	3.4 ± 35.4	−42–124.7	−2.64 [−12.9–10.5]	−35.2 ± 81.19	−377.4–11.8	−12.16 [−37.5–−3.8]	98	0.02
IIIA	0.3 ± 0.7	0–1.3	0.2 [−0.3–0.9]	−0.5 ± 1.06	−2.1–0.4	−0.06 [−1.6–0.3]	8	0.46
IIIB	0 ± 0.5	−1–0.6	0.05 [−0.4–0.4]	−0.6 ± 0.7	−1.7–0	−0.49 [−1.1–0]	6	0.24
IIIC	−0.6 ± 0.5	−1–−0.2	−0.54 [−0.9–−0.3]	−1.6 ± 1.13	−3.2–0	−1.55 [−2.4–−1]	5	0.17
IIID	−14.6 ± 21.7	−40–8	−12.95 [−32.1–2.9]	−31.5 ± 28.33	−79.3–0.5	−33.3 [−37.5–−5.9]	8	0.46

I: 1-month follow-up; III: 3-month follow-up. A. Length; B. Width; C. Depth; D. Volume.

**Table 13:** Relationship between patient age and treatment effects according to ultrasound measurements.

	Age						*U*	*p*
	<45 years			>45 years				
	M ± SD	Min–Max	Me [Q1–Q3]	M ± SD	Min–Max	Me [Q1–Q3]		
IA	−0.3 ± 0.8	−2–1.48	−0.4 [−0.7–0]	−0.1 ± 0.8	−2–1.5	−0.2 [−0.5–0]	150.5	0.42
IB	−0.3 ± 1	−2–1.9	−0.4 [−0.5–0.13]	−0.5 ± 1.1	−5–0.93	−0.33 [−0.7–0]	160	0.6
IC	−0.4 ± 0.7	−2–1.3	−0.5 [−0.8–−0.19]	−0.6 ± 0.9	−3–1.5	−0.46 [−0.9–−0.2]	162	0.64
ID	−6.9 ± 41.1	−75–124.7	−4.9 [−20–−2.43]	−26.8 ± 82.2	−377–17.81	−4.75 [−23.7–−1.04]	175	0.93
IIIA	0.4 ± 0.6	0–1.28	0.2 [−0.1–0.84]	−0.6 ± 1.1	−2–0.5	−0.25 [−1.6–0.3]	6	0.24
IIIB	0.1 ± 0.4	0–0.56	−0.1 [−0.2–0.3]	−0.6 ± 0.7	−2–0.3	−0.7 [−1.1–0.03]	5.5	0.2
IIIC	−0.8 ± 0.7	−1–0.02	−0.9 [−1.3–−0.23]	−1.5 ± 1.1	−3–−0.2	−1.36 [−2.4–−0.6]	7	0.34
IIID	−27.8 ± 40.4	−79–7.99	−19.9 [−59.9–4.26]	−22.6 ± 15.2	−37–−2.2	−27.89 [−34.5–−5.88]	12	0.92

I: 1-month follow-up; III: 3-month follow-up. A. Length; B. Width; C. Depth; D. Volume.

**Table 14:** Relationship between body weight assessment and treatment effect based on ultrasound measurements.

	Body Weight Assessment						*U*	*p*
	Normal			Abnormal				
	M ± SD	Min–Max	Me [Q1–Q3]	M ± SD	Min–Max	Me [Q1–Q3]		
**IA**	−0.02 ± 0.76	−1.5–1.31	0 [−0.6–0.5]	−0.3 ± 0.8	−2–1.5	−0.35 [−0.6–−0.1]	123	0.2
**IB**	−0.35 ± 1.42	−4.8–1.9	−0.33 [−0.6–0.3]	−0.4 ± 0.7	−2–1.16	−0.35 [−0.6–0]	153	0.72
**IC**	−0.55 ± 1.01	−3.2–1.3	−0.46 [−1–−0.05]	−0.4 ± 0.6	−2–1.5	−0.45 [−0.8–−0.19]	156	0.79
**ID**	−22.94 ± 104.95	−377.4–124.7	−4.91[−24.7–10.47]	−12.7 ± 20.9	−75–18.21	−4.71 [−20–−2.43]	154	0.75
**IIIA**	−0.72 ± 1.06	−2.1–0.3	−0.1 [−1.6–−0.09]	0.4 ± 0.6	0–1.28	0.4 [0–0.5]	4	0.09
**IIIB**	−0.76 ± 0.7	−1.7–0.03	−0.81 [−1.1–−0.2]	0 ± 0.4	−1–0.56	0.03 [−0.2–0.3]	3.5	0.07
**IIIC**	−1.92 ± 0.89	−3.2–0.96	−1.76 [−2.4–−1.3]	−0.5 ± 0.5	−1–0.02	−0.47 [−0.6–−0.2]	2	0.04
**IIID**	−30.07 ± 13.88	−40.4–−5.88	−34.51 [−37.5–−32.08]	−19.3 ± 35.5	−79–7.99	−2.2 [−23.7–0.53]	6	0.21

I: 1-month follow-up; III: 3-month follow-up. A. Length; B. Width; C. Depth; D. Volume.

**Table 15:** Relationship between history of delivery and treatment effect based on ultrasound measurements.

	Delivery						*U*	*p*
	Yes			No				
	M ± SD	Min–Max	Me [Q1–Q3]	M ± SD	Min–Max	Me [Q1–Q3]		
IA	−0.2 ± 0.8	−2–1.5	−0.35 [−0.64–−0.02]	−0.2 ± 0.81	−2–1.48	−0.1 [−0.6–0]	141.5	0.43
IB	−0.5 ± 1.1	−5–0.93	−0.32 [−0.6–0.17]	−0.3 ± 1.08	−2–1.9	−0.5 [−0.9–0]	163.5	0.9
IC	−0.5 ± 0.8	−3–1.5	−0.48 [−0.79–−0.19]	−0.4 ± 0.77	−2–1.3	−0.5 [−1–−0.07]	154.5	0.69
ID	−28.3 ± 77.7	−377–17.81	−4.96 [−26.94–−1.47]	−0.1 ± 39.32	−42–124.7	−4.2 [−13.5–0]	143	0.46
IIIA	−0.5 ± 1	−2–0.5	−0.09 [−1.6–0.3]	0.5 ± 0.7	0–1.28	0.4 [−0.1–1.28]	5	0.25
IIIB	−0.6 ± 0.7	−2–0.3	−0.6 [−1.12–0.03]	0.1 ± 0.43	0–0.56	−0.2 [−0.2–0.56]	6	0.36
IIIC	−1.3 ± 1.2	−3–0.02	−0.96 [−2.4–−0.2]	−1 ± 0.49	−1–0.47	−1.3 [−1.3–−0.47]	10	1
IIID	−19.3 ± 16.4	−37–0.53	−23.7 [−34.51–−2.2]	−37.2 ± 43.73	−79–7.99	−40.4 [−79.3–7.99]	7	0.49

I: 1-month follow-up; III: 3-month follow-up. A. Length; B. Width; C. Depth; D. Volume.

**Table 16:** Relationship between body weight assessment and bleeding duration.

Bleeding Duration	Follow-Up/Body Weight Assessment					
	Before		1 Month		3 Months	
	Normal	Abnormal	Normal	Abnormal	Normal	Abnormal
0	6 (30%)	12 (50%)	15 (100%)	20 (90.91%)	5 (100%)	3 (60%)
1	2 (10%)	0 (0%)	0 (0%)	2 (9.09%)	0 (0%)	2 (40%)
4	0 (0%)	1 (4.17%)	-	-	-	-
5	7 (35%)	2 (8.33%)	-	-	-	-
6	1 (5%)	3 (12.5%)	-	-	-	-
7	2 (10%)	6 (25%)	-	-	-	-
8	2 (10%)	0 (0%)	-	-	-	-
Chi^2^ NW	***χ*^2^ = 14.69**	***p* = 0.02**	*χ*^2^ = 2.16	*p* = 0.14	*χ*^2^ = 3.28	*p* = 0.07

## Data Availability

The data presented in this study are available on request from the corresponding author.
